# Parental Leave and Fertility: Individual-Level Responses in the Tempo and Quantum of Second and Third Births

**DOI:** 10.1007/s10680-023-09669-0

**Published:** 2023-07-05

**Authors:** Allan Puur, Sanan Abdullayev, Martin Klesment, Mark Gortfelder

**Affiliations:** Tallinn University, Estonian Institute for Population Studies, Tallinn, Estonia

**Keywords:** Parental leave, Second and third births, Speed premium, Cure model, Estonia

## Abstract

Previous studies have documented varying fertility responses to changes in parental leave provisions. We contribute to this literature by investigating the effects on the transition to second and third births of a policy reform that introduced generous earnings-dependent parental leave benefit in Estonia in 2004. Our study employs a mixture cure model, a model with some useful properties that has been seldom applied in fertility research. The advantage of the cure model over conventional event history models is the ability to distinguish the effect of the covariates on the propensity to have a next child from their effect on the tempo of childbearing. The results show that the transition to next birth accelerated as parents responded to so-called speed premium, a feature that allowed them to avoid a reduction in benefits caused by a reduction of earned income between births, through the closer spacing of births. Furthermore, the findings suggest that the introduction of generous earning-related parental leave was associated with a substantial increase in the progression to both second and third births.

## Introduction

Sub-replacement fertility has persisted in developed nations for already several decades. From 1986 to 2015, the number of countries that report the aim to raise fertility nearly tripled (United Nations, 2018). In order to stimulate birth rates, governments are turning to family policies that have become indispensable parts of the modern welfare state (Neyer, 2013; Rindfuss & Choe, 2016; Thévenon, 2011). The increasing importance of family policies is evident from the resources allocated to them. Since 1990, in OECD countries, public spending on family policies increased from 1.5 to 2.3% of GDP, with the growth rate being faster than that of social expenditures as a whole (OECD, 2022).

Among family policy measures, parental leave is an important instrument aimed at providing employment security so that parents can take time off work to care for a young child, with a guaranteed return to their job when the leave ends. In an increasing number of countries, job-protected parental leave goes in hand with benefits that compensate for individual income lost during the employment break (Koslowski et al., 2020). Parental leave bridges the gap between the birth of a child and its entry into formal childcare, if childcare services are widely available. Furthermore, if a certain proportion of parental leave is reserved for fathers, parental leave can foster more a gender-equal division of childcare (Karu & Tremblay, 2018; Koslowski 2021).

These considerations, supported by the understanding that people have more children when costs of childrearing are lower (Becker, 1991; Hotz et al., 1997), lead to the expectation that generous parental leave provisions contribute to higher fertility. However, several frequently cited reviews remain inconclusive on the effects of parental leave benefit on the fertility level, describing them as ʻweak’, ʻmixed’ or ʻinconsistent’ (Gauthier, 2007; Sleebos, 2003; Sobotka et al., 2020). While several empirical studies have shown that generous parental leave provisions produce tempo-effects that push the period fertility rates temporarily upwards (Andersson, 1999, 2004; Andersson et al., 2006; Hoem, 1990, 1993; Miranda, 2020), the evidence of the quantum effects of parental leave on fertility is less coherent (Dahl et al. 2016; Cannonier, 2014; Lalive & Zweimüller, 2009; Št’astná & Sobotka, 2009; Št’astná et al. 2020).

This study expands the literature on individual-level fertility responses to family policy measures by analysing the shift to a more generous earnings-related parental leave. Our aim is to provide evidence on the changes in the tempo and quantum of second- and third-order fertility, associated with the parental leave reform in Estonia (Puur & Klesment, 2011). Examining the impact of this reform is of scholarly interest for several reasons. First, the largely unanticipated introduction of the reform in January 2004 is a clear break with the previous system. Second, in international comparisons, Estonia stands out for the generosity of its parental leave system, topping the list in several measures (Otto et al., 2021). Third, the time passed since the reform allows for analysing both its short- and long-term effects. Finally, the longitudinal microdata from registers allows for comparing the fertility behaviour of women before and after the parental leave reform, controlling for a variety of individual characteristics and macroeconomic conditions. We believe that the above features provide a basis for a reliable assessment of the effects of the policy change.

Our study makes two contributions to the literature. The first contribution stems from the use of the mixture cure model, which, unlike standard event history models, is capable of separating the effect of the explanatory variables on the propensity of having another child from their effect on its timing. The importance of this capacity can hardly be overstated as the impact of policy changes is often limited to the timing of childbearing (Sobotka et al., 2020). To the best of our knowledge, the mixture cure models have not been used for the study of family policy reforms. Second, this study contributes to a more comprehensive account of the effects of parental leave reforms on fertility by extending the evidence beyond a small number of countries that have been the focus of previous research.

## Parental Leave and Its Effects on Fertility

The understanding that policies can stimulate fertility draws on the microeconomic theory of the family, frequently used as a framework for analyses of family policy reforms. The main presumption of the microeconomic theory is that the number of children couples choose to have depends on their purchasing power, resources expected to be needed for rearing children, and parents’ preferences for spending these resources on children rather than using them for other purposes (Becker, 1991; Cigno, 1994). In this framework, policy changes that increase family income are expected to lower the direct costs of rearing a child, or both, should translate into higher fertility, unless parents decide to trade quantity for quality of offspring (Becker & Lewis, 1973). By contrast, policy changes that strengthen attachment of parents to the labour force and/or increase wages, tend to increase the opportunity costs due to time off work to care for children. In this context, parental leave schemes with an employment guarantee and benefits that compensate for foregone earnings are seen to reduce both the opportunity costs and the direct costs of having children (Bergsvik et al., 2021; Matysiak & Szalma, 2014).^1^

Studies which apply an aggregate-level approach have found that the association between the parental leave entitlements on the total fertility rate ranges from positive (Adserà, 2004; Luci-Greulich & Thévenon, 2013) to insignificant (Gauthier & Hatzius, 1997; Hilgeman & Butts, 2009) to negative (D’Addio & d’Ercole, 2005). It has been argued that negative or insignificant estimates might stem from a failure to control for the availability of care services, but this explanation does not apply for all studies mentioned above. A few aggregate-level studies have investigated whether more generous parental leave payments are associated with higher fertility, net of tempo distortions, but these results have also been mixed. Luci-Greulich and Thévenon (2013) found that longer fully paid parental leave in OECD countries is associated with a small increase in the tempo-adjusted TFR. By contrast, for Western European countries, Kalwij (2010) showed that an increase in maternity and parental leave benefits could reduce childlessness, but would have no significant effect on completed family size. A similar finding was reported by Baizan et al. (2016) for 15 countries of the European Union.

There is a growing understanding that reliable assessment of the effects of family policies requires research designs that are based on longitudinal microdata and appropriate statistical techniques (Bergsvik et al., 2021; Neyer & Andersson, 2008). Unlike studies based on aggregate measures, these approaches allow researchers to zoom in on the connection between policy variation and people’s actual fertility behaviour. Pioneering studies into the individual-level effects of parental leave on fertility behaviour, based on register data and using event history methods, were conducted in Sweden, a country with a long tradition of well-paid parental leave determined by the parents’ pre-leave earnings.

Much of the Swedish research has focused on the effects of so-called speed premium, a special feature of the parental leave, which guarantees parents the amount of benefit they received before the previous births if subsequent births are spaced sufficiently closely (Carlson, 2013; Duvander, 2008). Several analyses have shown that extending the eligibility interval for speed premium to 30 months in 1986 resulted in a marked change in birth spacing behaviour, reflected in the increase in second- and third-birth rates at short durations (Andersson, 1999, 2002, 2004; Hoem, 1990, 1993; Miranda, 2020). These findings are considered evidence of a causal link between the policy change and the shortening of birth intervals (Bhrolcháin & Dyson, 2007). The shift towards shorter birth intervals was the main factor behind the marked increase in the period total fertility rate in Sweden in the 1980s (Neyer & Andersson, 2008). However, studies on Sweden have not addressed long-term changes in fertility quantum associated with parental leave. In the Nordic context, scant evidence for such a change comes from Norway. Using a regression discontinuity design, Dahl et al. (2016) showed that the extension of earnings-related parental leave in 1992 had a marginally positive effect on women’s number of children 14 years later. The small effect size may be due to the fact that the studied extension of leave spanned only three weeks.

In Austria, a policy reform in 1990 increased the duration of job-protected paid parental leave from 12 to 24 months; the parents could renew their leave if the next child was born during the leave period, with a short additional bridging period (Thenner, 1999). Several studies showed that the policy change resulted in a marked increase of second- and third-birth rates at short durations, as well as in shortening birth intervals (Prskawetz & Zagaglia, 2005; Prskawetz et al., 2008; Št’astná & Sobotka, 2009). Furthermore, Lalive and Zweimüller (2009), who applied a regression discontinuity approach, found that the effect of the reform was not limited to tempo. Among women eligible for the more generous leave, three additional children per 100 women were born within ten years after the first birth, compared to their counterparts who were not eligible. The evidence for the quantum effect was questioned by Št’astná and Sobotka (2009), but reliance on descriptive methods gives less weight to their findings.

In Germany, a parental leave reform in 2007 replaced means-tested flat-rate benefits aimed at low-income groups with a universal earnings-related scheme. As part of the reform, the duration of leave was reduced from 24 to 12 months to facilitate mothers’ faster return to work (Spiess & Wrohlich, 2008). Using a combination of regression discontinuity design and a difference-in-differences approach, Cygan-Rehm (2016) showed that fertility response varied across time and socioeconomic groups, in line with the complex change in the incentive structures. With regard to the tempo of childbearing, fertility rates decreased at shorter durations, and the intervals between births increased. However, except for low-income mothers, who were worse off after the reform, the negative effect of the reform became insignificant at the fifth year since previous birth. These findings are corroborated by Raute (2019), who demonstrated that the reform resulted in a marked increase in the probability of having a next child among tertiary-educated women, and women with medium and high incomes. However, as the observation ended five years after the reform, the results of German studies are inconclusive regarding the long-term (level) effect.

There are two important studies from North America that employ a difference-in-differences design. In 2006, Quebec significantly increased the generosity of parental leave benefits, while no similar policy change occurred in other provinces. Based on the Canadian Labor Force Survey, Ang (2015) estimated that the reform resulted in a substantial increase in fertility rate in Quebec relative to the rest of country. However, the short observation period after the reform (up to 2008) did not allow the author to distinguish the tempo and quantum effects. In the USA, a federal reform in 1993 introduced 12 weeks of job-protected unpaid leave to employees who met the eligibility criteria. Using the National Longitudinal Survey of Youth (NLSY), Cannonier (2014) showed that the reform was associated with both the tempo and quantum effects: eligible women increased the probability of first and second births, and had a next child sooner than their non-eligible counterparts. A potential limitation of this study stems from the fact that it includes only one NLSY cohort that had reached peak fertility at the time of the policy change.

A few studies have sought evidence of the effects of parental leave on fertility by comparing countries that share similarities in their demographic, economic, and cultural characteristics, but differ in their parental leave arrangements. Using the Generations and Gender Survey, Matysiak and Szalma (2014) associated higher second conception hazard in Hungary with universal earnings-related parental leave payments in that country, relative to Poland, where parents were entitled to low means-tested benefits. The authors hypothesised that the differences in parental leave provisions mainly affect the timing of childbearing, but their approach did not make it possible to give a firm answer to this question. By contrast, comparing second births in Slovakia and Czechia, Št’astná et al. (2020) showed that after an increase in the generosity and flexibility of parental leave, Czech women not only accelerated the transition to second birth, but also exhibited an increase in parity progression 10 years after first birth over their counterparts in Slovakia. However, as the authors rely on descriptive methods, the difference in fertility indicators between the two countries cannot, with certainty, be attributed to variation in parental leave arrangements.

To sum up, previous studies based on longitudinal microdata have shown that earnings-related parental leave, particularly when it involves so-called speed premium, leads to a shortening of birth intervals. However, there is less evidence as to whether generous earnings-related parental leave is associated with shifts in fertility quantum. In this article, we seek to complement the existing research using the mixture cure model and register-based data from Estonia.

## The Context

Since the 1990s, Estonia has developed towards a family policy model characterised by earning-related parental leave, universal family benefits, broad-coverage public childcare, and improving gender equity (Frejka et al., 2016). The total spending on family policies is rather generous, equalling or slightly exceeding 3% of the GDP in recent years. Currently, enrolment in childcare among children aged 1 and 2 are 33% and 74%, respectively; in the age group 3–5, it exceeds 90% (Statistics Estonia 2022). The employment rate of Estonian women ranks near the top among the European Union countries, with a very small gender gap (Eurostat, 2022).

Universal job-protected parental leave was extended to three years in 1989, but the income compensation remained low until the scheme was revised in 2004 (Katus et al., 2004). The reform replaced low flat-rate benefits with earnings-related benefits, amounting to the full salary of the calendar year prior to childbirth (Puur & Klesment, 2011). The maximum amount was set at three average salaries; parents who had not worked during the previous year were entitled to a flat-rate benefit slightly below the minimum wage. Initially, the duration of parental benefit was 11 months following childbirth; in 2006, it was expanded to 14 months, and in 2008 to 18 months. From 2004, if the interval between successive births does not exceed 30 months, the parents could retain the same level of benefits which they were entitled to before the previous birth.

From 2007, fathers could take parental leave right after the end of maternity leave (when the child is 70 days old). Since then, the proportion of fathers among benefit recipients has slowly increased, but the leave is still mainly used by mothers; in 2021 women constituted 84% of benefit recipients. Between 2018 and 2022, further changes were made to the system with the aim of increasing the flexibility of leave use and the proportion of fathers among leave takers (Biin, 2017; Social Insurance Board, 2022). Due to the combination of full income compensation, high maximum amount, and long duration, the Estonian parental leave scheme is one of the most generous in the world. It ranks first in terms of the percentage of GDP allocated to the scheme, and second in terms of public expenditure per one live birth (Otto et al., 2021).

The leave expansion coincided with the end of very low fertility in Estonia that had lasted nearly a decade since mid-1990s. After the reform, the total fertility rate showed an upward trend for several years: it increased from 1.37 in 2003 to 1.72 in 2010, declining only after the peak of economic recession. In the literature, the marked increase has been attributed to the parental leave reform (Goldstein et al., 2009, 2013), but to date, the link between the reform and fertility behaviour has not been thoroughly investigated. An applied study commissioned by the Ministry of Social Affairs (Võrk et al., 2009) identified a shift towards shorter birth intervals, but descriptive methods prevented the authors from providing reliable evidence on the effect on fertility quantum. The same limitation applies to the discussion of the Estonian parental leave reform in a recent UNFPA report on the effects of family policies in low-fertility settings (Sobotka et al., 2020).

## Research Hypotheses

We formulate three hypotheses on the association between the Estonian parental leave reform and second- and third-birth spacing and quantum of fertility.

Our first hypothesis (H1) posits that the speed premium in tandem with the long duration of paid parental leave would be associated with faster progression to second and third births. The hypothesis draws on the notion that the possibility of retaining the previous level of benefits without returning to work in between closely spaced births is an option attractive to many parents.

Our second hypothesis (H2) anticipates that the introduction of well-paid parental leave would lead to an increase in the quantum of childbearing. This expectation stems from the microeconomic theory of the family, according to which parents could have more children when the costs of childrearing would be lower.

Our third hypothesis (H3) expects that the changes in fertility behaviour associated with parental leave reform would be more pronounced in the transition to the second birth. As regards fertility quantum, our expectation is based on the dominance of two-child families that has been as an intrinsic characteristic of low-fertility societies (Frejka, 2008), in which two children have been both the preferred and the ideal family size (Beaujouan & Berghammer, 2019; Bongaarts, 2002; Sobotka & Beaujouan, 2014). For parity progression past the second child, having another child can be seen more as a matter of individual preference (Ryder, 1980), for which parental leave arrangements may be less important. Furthermore, our hypothesis draws on the assumption that the second-birth rates are not at maximum levels, and there is substantial unmet demand for second births in the study population.^2^ As regards the timing of childbearing, the unmet demand for second births and the higher fecundity of women with one child, which is associated with their younger age, leads us to expect that the shortening of birth intervals is more pronounced for second births.

## Data, Methods and Variables

Our study is based on longitudinal register data compiled by Statistics Estonia. The dataset contains childbearing and marital histories of women born between 1960 and 1990, women’s and their partners’ socio-demographic characteristics, measured at the beginning of each birth interval starting from 1993, and censoring events.

The events investigated are the conception of the second and third child, backdated from live births. The exposure time, measured with monthly accuracy, starts at the birth of the first (second) child and ends at the conception of the next child; included in the study are birth intervals that start between 1993 and 2019. Observations are censored at women’s 45th birthday, death, emigration, or at the end of observation period (31 March 2019).^3^ The analytical dataset includes 125,284 women for the analysis of second births, and 89,936 women for third births.

To investigate the association between the policy reform and childbearing behaviour, we use descriptive methods and event history models. Our analysis begins with duration-specific rates and Kaplan–Meier estimates. This is followed by mixture cure models, also known as split-population models, which is an extension of conventional survival models (Berkson & Gage, 1952; Boag, 1949). The cure models comprise two submodels, one (incidence submodel) focusing on quantum, and the other (duration submodel) on tempo of the process. In the context of fertility research, the main advantage of the mixture cure model over conventional event history models is the ability to distinguish the effect of covariates on the risk of progressing to the next child from their effect on the timing (Bremhorst et al., 2016; Gortfelder & Puur, 2020; Gray et al., 2010).

For estimation, we used ʻcureregr’ in Stata 14 (Buxton, 2013). Our submodels estimate the effects of same covariate vectors of ${z}_{i}$ and ${x}_{i}$ on risk of progressing from first (second) birth to the next birth, and on its timing, respectively. The model assumes that women who had a first (second) birth are composed of two groups: i) a group that experiences the next birth with a probability of $\pi$, and ii) a group that does not experience it, with a probability of $1-\pi$. In the incidence submodel, the risk of progressing to the next child is modelled with logistic function. The duration submodel is an accelerated failure time (AFT) model that estimates the multiplicative effects of covariates on time T for those who progress to the next parity; a lognormal distribution is used for the shape of hazard rate. The log-likelihood function of the model is expressed as:

$$l\left(\theta \right)=\mathrm{log}{\prod }_{i=1}^{n}{\left[\pi \left({z}_{i}\right){f}_{u} \left({t}_{i }\right| {x}_{i})\right]}^{{\delta }_{i}}{\left[1-\pi \left({z}_{i}\right)+ \pi \left({z}_{i}\right){S}_{u }\left({t}_{i }\right| {x}_{i})\right]}^{1-{\delta }_{i}}$$

The notations of ${f}_{u} \left({t}_{i }\right| {x}_{i}$) and ${S}_{u }\left({t}_{i }\right| {x}_{i}$) are failure and survival rates, respectively. While interpreting the estimates from incidence model, a positive or negative coefficient indicates increasing or decreasing risk of an event. In duration model, they are interpreted as the lengthening (positive) or shortening (negative) of birth intervals.

Our main explanatory variable is a time-varying indicator variable that equals one if a woman is at risk of conception of a second or third birth in the period after the reform (from 1 January 2004 to 31 March 2019), and zero if the exposure falls within the period preceding the reform (from 1 January 1993 to 31 December 2003). To account for changes in the composition of the population at risk and macroeconomic conditions, we included a number of control variables in the models. The choice of controls was guided by previous studies, as well as data limitations. Individual controls included woman’s age group, partnership and marital status, education, occupational/activity status, ethnicity, area of residence, birth cohort, sex of previous children, and male partner’s education. Aside from marital status, which is a time-varying covariate, other individual controls were measured at the beginning of birth intervals. Controls for macroeconomic conditions included the GDP growth rate, the unemployment rate, and the consumer confidence index. All macroeconomic controls are time-varying, measured on an annual basis. Descriptive statistics for the explanatory and control variables are presented in Table 1.

**Table 1:** Descriptive statistics for the explanatory and categorical control variables by parity progression, women born 1960–1999, Estonia

Variables	Parity progression							
	1 → 2				2 → 3			
	Exposure		Events		Exposure		Events	
	Person-months	%	Second births	%	Person-months	**%**	Third births	%
*Policy indicator*								
Before reform	3,012,249	28.2	22,670	26.3	2,202,370	23.7	7066	23.7
After reform	7,677,577	71.8	63,535	73.7	7,084,603	76.3	22,706	76.3
*Woman’s age at previous birth*								
Under 20	1,705,384	15.9	15,394	17.9	133,633	1.4	1141	3.8
20–24	4, 640,298	43.4	37,807	43.9	2,002,698	21.6	9592	32.2
25–29	3,023,749	28.3	25,149	29.2	3,938,474	42.4	12,047	40.5
30–34	1,053,219	9.9	6804	7.9	2,514,490	27.1	5835	19.6
35–39	246,542	2.3	1003	1.2	650,608	7.0	1113	3.7
40–44	20,634	0.2	48	0.1	47,070	0.5	44	0.2
*Woman’s birth cohort*								
1960–1964	222,350	2.1	532	0.6	490,239	5.3	550	1.9
1965–1969	864,549	8.2	3811	4.4	1,835,673	19.8	3291	11.1
1970–1974	2,663,084	24.9	16,462	19.1	3,108,082	33.5	8181	27.5
1975–1979	3,060,943	28.6	24,998	29.0	2,214,954	23.6	8851	29.7
1980–1984	2,273,925	21.3	22,811	26.5	1,206,587	13,0	6045	20.3
1985–1989	1,265,179	11.8	13,705	15.9	380,138	4.1	2413	8.1
1990–1994	314,771	2.9	3575	4.2	50,355	0.5	427	1.4
1995–1999	25,025	0.2	311	0.4	945	0.0	14	0.6
*Woman’s partnership status at previous birth*								
Married	3,725,986	34.8	30,943	35.9	5,329,493	57.4	14,881	50.0
Cohabiting	5,503,479	51.5	46,883	54.4	3,639,238	39.2	13,617	45.7
Not partnered	1,455,380	13.6	8338	9.7	313,184	3.4	1262	4.2
Unknown	4981	0.1	41	0.1	5058	0.1	12	0.0
*Woman’s marital status (time-varying)*								
Married	3,605,537	33.7	36,114	41.9	5,301,474	57.1	15,717	52.8
Never married	6,186,755	57.9	45,892	53.2	3,010,589	32.4	11,409	38.3
Formerly married	869,514	8.1	3831	4.4	947,050	10.2	2473	8.3
Re-married	28,020	0.3	368	0.4	27,860	0.3	173	0.6
*Sex of previous child(ren)*								
Boy	5,462,608	51.1	44,604	51.7	–	–	–	–
Girl	5,227,218	48.9	41,601	48.3	–	–	–	–
Mixed	–	–	–	–	4,721,297	50.8	13,880	46.6
Both boys	–	–	–	–	2,443,099	26.3	8550	28.7
Both girls	–	–	–	–	2,122,577	22.9	7342	24.5
*Woman’s education at previous birth*								
Low (ISCED0–2)	1,663,743	15.6	15,361	17.8	847,732	9.1	5182	17.4
Medium (ISCED3–4)	6,762,151	63.3	47,883	55.6	6,086,767	65.5	17,019	57.2
High (ISCED5–8)	2,242,105	21	22,820	26.5	2,337,130	25.2	7527	25.3
Unknown	21,827	0.2	141	0.2	15,344	0.2	44	0.2
*Male partner’s education at previous birth*								
Low (ISCED0–2)	1,343,714	12.6	12,390	14.4	804,847	8.7	4348	14.6
Medium (ISCED3–4)	6,762,428	63.3	51,311	59.5	6,325,370	68.1	18,108	60.8
High (ISCED5–8)	1,636,251	15.3	17,278	20.0	1,892,537	20.4	6304	21.2
Unknown	947,433	8.9	5226	6.1	264,219	2.9	1012	3.4
*Woman’s occupation/activity status at previous birth*								
Upper white collar	1,633,564	15.3	16,201	18.8	1,832,618	19.7	5707	19.2
Lower white collar	3,880,902	36.3	28,929	33.6	3,521,635	37.9	9433	31.7
Manual occupation	1,383,780	12.9	9265	10.8	1,370,047	14.6	3958	13.3
Occupation unknown	241,808	2.3	2748	3.2	238,011	2.6	791	2.7
Unemployed	651,842	6.1	4622	5.4	503,780	5.4	1725	5.8
Homemaker	1,538,369	14.4	12,258	14.2	1,614,012	17.4	7085	23.8
Student	1,306,673	12.2	11,869	13.8	173,693	1.9	926	3.1
Other	45,397	0.4	243	0.3	26,655	0.3	119	0.4
Unknown	7491	0.1	70	0.1	6522	0.1	28	0.1
*Woman’s ethnicity*								
Estonian	6,908,185	64.6	64,088	74.3	6,741,711	72.6	24,065	80.8
Other ethnic groups	3,781,641	35.4	22,117	25.7	2,545,262	27.4	5707	19.2
*Woman’s area of residence at previous birth*								
Capital	3,986,292	37.3	29,138	33.8	2,766,834	29.8	7702	25.9
*Areas surrounding*								
the capital	525,449	4.9	5235	6.1	623,909	6.7	2094	7.0
Big towns	2,270,637	21.2	17,227	20.0	1,847,175	19.9	5274	17.7
Smaller towns and rural areas	3,868,762	36.2	34,413	39.9	4,026,354	43.4	14,653	49.2
Unknown	38,686	0.4	192	0.2	22,701	0.2	49	0.2
Number of women	125,284				89,936			

The observation window starts on 1 January 1993 and ends 31 March 2019

*Source:* Statistics Estonia, authors’ calculations

## Results

### Duration-Specific Fertility Rates

As a first step, we examine trends in duration-specific second- and third-birth rates for parity cohorts of women who gave their previous birth in the period from 1995 to 2015.

**Fig. 1:**
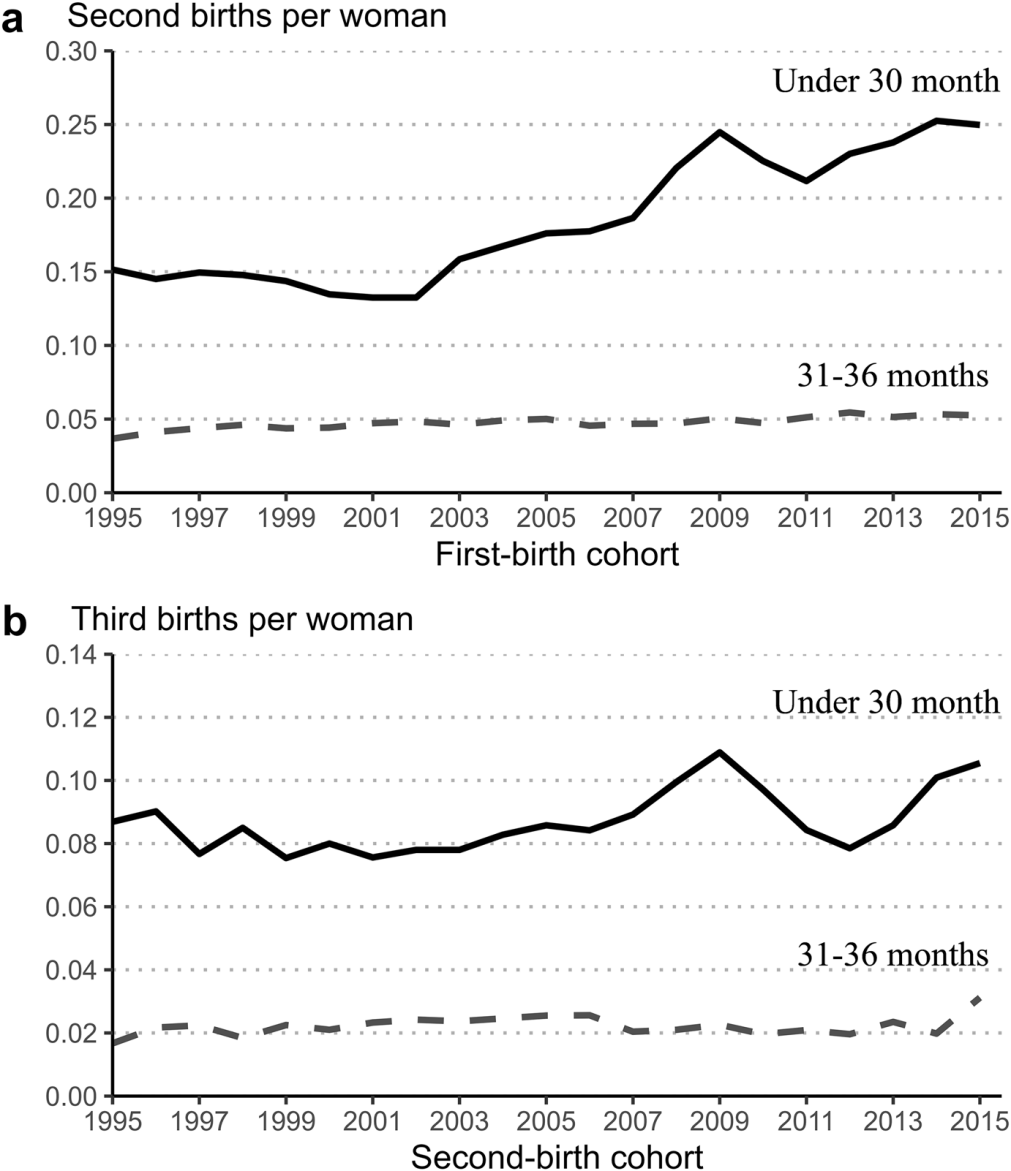
Duration-specific second- and third-birth rates (short durations), female parity cohorts 1995–2015, Estonia. *Source:* Statistics Estonia, authors’ calculations

At duration under 30 months, there is a noticeable increase in the progression to second birth, starting from women who had their first child in 2003 (panel a of Fig. 1). The increase persists over several parity cohorts, peaking at the 2009 cohort. In that cohort, the second-birth rate during the first 30 months of the birth interval exceeds the baseline cohort (2002) by 1.85 times. By contrast, at duration from 31 to 36 months, the progression to next child remains virtually unchanged in the same cohort range. A largely similar pattern can be observed for third births, although the increase is less pronounced (panel b of Fig. 1). From the 2002 to 2009 cohort, the third-birth rate at duration under 30 months increased by 1.40 times.^4^

The increase in second- and third-birth rates at duration less than 30 months since the previous birth can be linked to the 2004 parental leave reform. In particular, two aspects of the results support this. First, the increase in fertility rates occurred precisely in the interval of eligibility for the speed premium, with no similar increase observed at a slightly longer duration of 31–36 months. Second, the increase in fertility rates under 30 months since the previous birth started from the parity cohorts exposed to the parental leave reform. Parents who had their previous child in 2003 were the first who had the opportunity to take advantage of the speed premium. When the reform came into force, parents who had their previous child in early 2003 had one and a half years to have a next child within the period of eligibility. By contrast, this was not possible for their counterparts who had a child at the beginning of 2002.

Another aspect of the results that deserves attention is the gradual nature of the increase in fertility rates under 30 months. If it was caused only by the speed premium that came into force in January 2004, then why did the full effect of the speed premium take about five years (parity cohorts) to build up? We believe that the explanation must be sought mainly from two factors. The first is the dependence of parental leave benefits on the income earned before childbirth. Võrk et al. (2009) showed that female employment and wages during the year preceding childbirth markedly increased after the reform as women increased their labour market attachment in order to benefit more from the new system. However, the adaptation did not happen instantly, but took several years. Another plausible contributing factor was the extension of the payment period of parental leave benefits from the initial 11 months to 14 months in 2006, and further to 18 months in 2008. For parents who were considering having a next child after a short interval, the extension of the payment period reduced the risk of experiencing several months with very low income. The extension of the payment period also reduced the need to return to work for only a very brief period, thus making close spacing of births more attractive for parents.

**Fig. 2:**
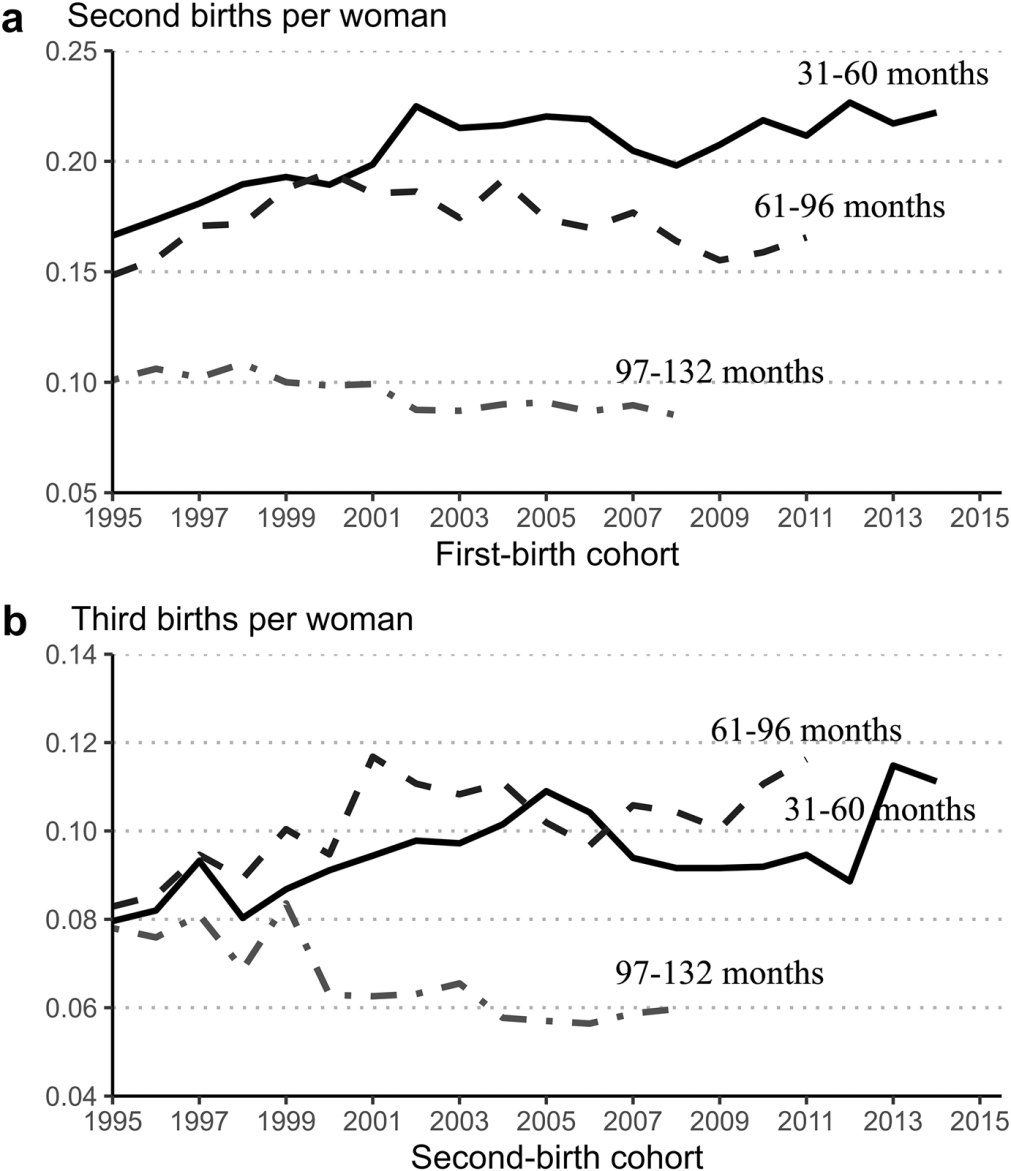
Duration-specific second- and third-birth rates (longer durations), female parity cohorts 1995–2015, Estonia. *Source:* Statistics Estonia, authors’ calculations

Figure 2 shows the second- and third-birth rates at durations beyond the period of eligibility for speed premium. To detect a link between the policy reform and the change in fertility rates at these longer durations, attention must be focused on parity cohorts from which exposure to the reform begins in each duration interval considered. Concentrating on these cohorts is equivalent to examining ʻcritical junctures’, or time periods during which the policy changed (Neyer & Andersson, 2008).

In the duration interval from 31 to 60 months, the exposure to the parental leave reform begins with the 1999 parity cohort. Parents who had a child born at the end of 1999 had experienced four years since the previous birth by the time of the reform in January 2004. This means that the members of this parity cohort were exposed to the reform only in the last year of the interval from 31 to 60 months. In the 2000 and 2001 parity cohorts, the exposure to the reform started earlier, respectively. Finally, the 2002 cohort was influenced by the reform from the beginning of the given duration interval. Results in Fig. 2 show that between the 1998 cohort (the last cohort which was not exposed to the reform in the interval from 31 to 60 months) and the 2002 cohort, second- and third-birth rates in the interval in question gradually increased.

To provide insight into the connection between the parental leave reform and fertility change in interval from 61 to 96 months since the previous birth, attention must be focused on other parity cohorts. The earliest cohort, which was exposed to the reform at the end of the considered interval, comprises parents who had their previous child in 1996. In contrast, the 1999 parity cohort was the one influenced by the reform from the beginning of the interval. Similar to the results reported above for the interval from 31 to 60 months, exposure to the reform in the interval from 61 to 96 months is also associated with an increase in second- and third-birth rates. The observation window of our study does not make it possible to provide clear evidence on the association between changes in fertility rates and the parental reform at durations over 96 months.

### Kaplan–Meier Estimates

**Fig. 3:**
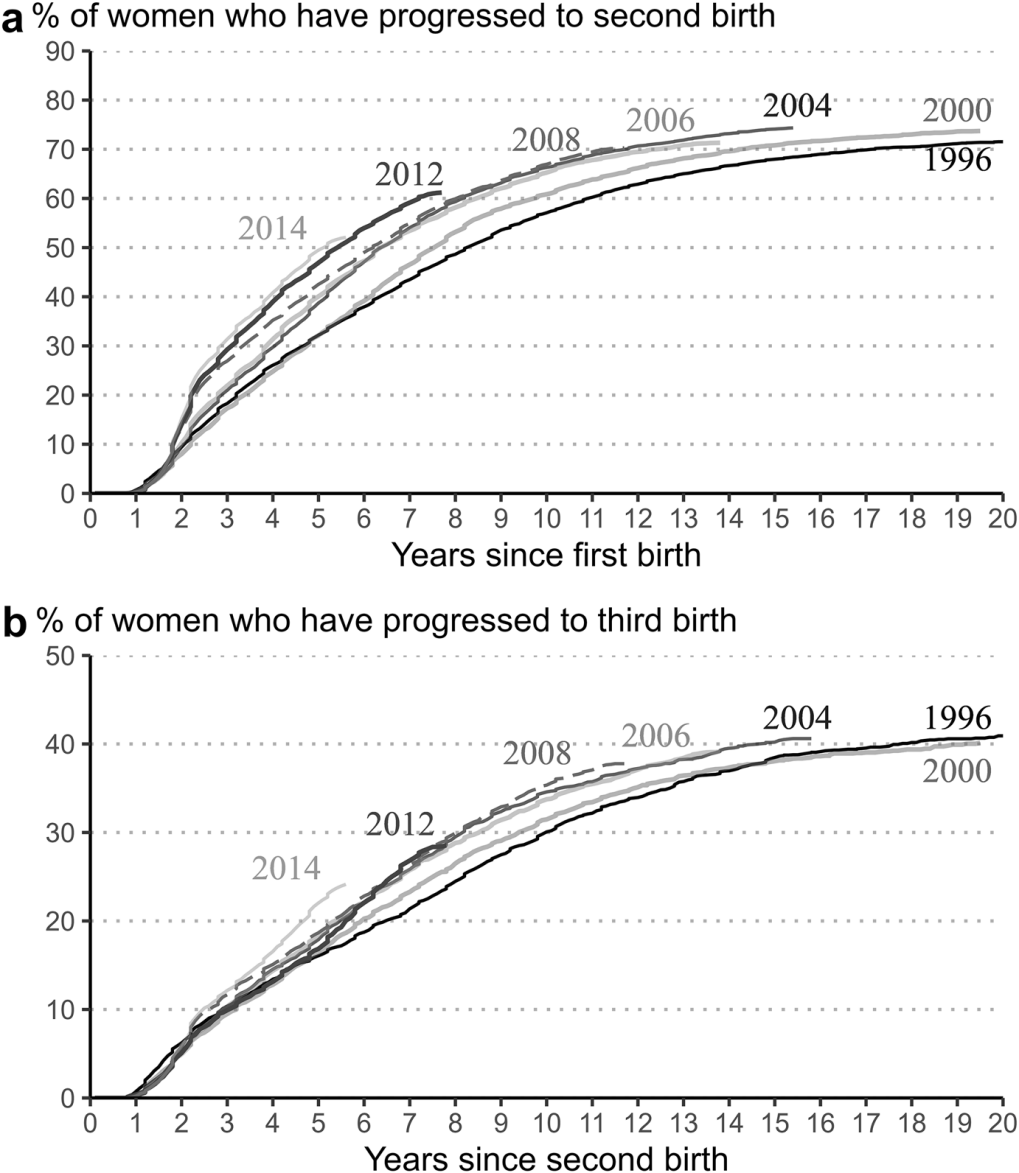
Inverted Kaplan–Meier estimates for transition to second and third birth, female parity cohorts 1996–2014, Estonia. *Source:* Statistics Estonia, authors’ calculations

Duration-specific fertility rates revealed an increase in second- and third-birth rates following the parental leave reform, especially at short durations since the previous birth. However, the evidence obtained from duration-specific rates is fragmented, and does not provide a clear picture of shifts in the quantum of fertility. For this purpose, we use inverted Kaplan–Meier curves to show the proportion of women who had their second or third child by time since previous birth (Fig. 3).^5^

We begin the examination from women who gave birth to their first child in 1996 and 2000, eight and four years before the policy reform, respectively (panel a of Fig. 3). The results show great similarity in women’s experiences in these parity cohorts at short durations. However, around the fifth year, when the 2000 cohort is exposed to the new parental leave scheme, the curves diverge. The difference between the two cohorts increases until the eighth year, and peaks at five percentage points. At longer durations, where the earlier cohort also becomes exposed to the reform, it decreases.

The 2004 and 2006 first-birth cohorts are among the first cohorts exposed to new system immediately after having a first child. Kaplan–Meier estimates show that the progression to second birth is very similar for these cohorts. As expectedly, the proportion of women who have a second child already starts to exceed that of the pre-reform cohorts at short durations. However, at the point where the speed premium expires, the difference between our early post-reform cohorts (2004 and 2006) and the baseline cohort (1996) is small. However, this difference increases at longer durations, and thus five years after the first birth, the proportion of women in the post-reform cohorts who have progressed to second birth exceeds that of the baseline cohort by 7–8 percentage points. In the seventh and eighth years of the policy reform, the difference reaches 10 percentage points. At longer durations, it decreases somewhat, as the baseline cohort became exposed to the new system as well.

The 2008 first-birth cohort follows a somewhat different pattern. At short durations, it exhibits a noticeably faster progression to second birth compared to the 2004 and 2006 cohorts, in line with the strengthening of the effect of speed premium. However, at longer durations, the progression to second birth slows down, plausibly due to the Great Recession, leading to convergence with early post-reform cohorts. In the 2012 and 2014 cohorts, the rapid progression to second birth during at short duration is even more pronounced. Rather than converging with early post-reform cohorts, the 2012 and 2014 first-birth cohorts demonstrate a noticeably higher progression to second birth until the end of observation.

The inverted Kaplan–Meier curves for progression to third birth show patterns that are largely similar to those for second births (panel b of Fig. 3). Similar to second births, the curves of the 1996 and 2000 parity cohorts diverge five years after the previous birth, when the later cohort is exposed to the parental leave reform. The difference increases until the seventh and eighth year, after which it begins to narrow, as the 1996 cohort becomes exposed as well. At very long durations, the proportion of women in the 1996 cohort who gave birth to a third child catches up with that of the 2000 cohort.

A comparison of the pre-reform (1996 and 2000) and post-reform (2004 and later) cohorts leads to similar findings regarding second births, with the exception of smaller differences between cohorts. A higher progression to the next child in the post-reform cohorts over the pre-reform cohorts persists at longer durations. For instance, at the point where observation of the 2012 cohort ends (seven years after the second birth), the proportion of women who have given birth to a third child exceeds that of the baseline cohort (1996) by 5.5 percentage points. A further increase in the progression to the third birth can be observed in the 2014 cohort; this increase is not related to the parental leave reform, but to the 2017 reform of child benefits that favoured families with three or more children.

### Mixture Cure Models

#### Estimates for the Parental Leave Reform

To further investigate the link between the parental reform and fertility outcomes, we employ mixture cure models. The event of interest in the models is the conception of a second or third child. Our main explanatory variable compares the incidence and timing of conception of an additional child in the periods after and before the parental leave reform.^6^ For both parity transitions, we estimated a series of main effects models, and monitored the change in the effect of the main explanatory variable as controls were added in a stepwise manner.

Table 2 presents the exponentiated parameter estimates of our policy variable. Parameter estimates in the incidence submodel are presented in the form of odds ratios, which are interpretable same way as in the logistic regression. In all incidence submodels, the results reveal a positive association (odds ratios over 1) between the parental leave reform and the propensity of conceiving an additional child. The odds ratio decreases after adding controls for mother’s characteristics to the model (M2). This suggests that changes over time in these characteristics are partly responsible for the increase in propensity of additional birth in the period after the policy change. However, despite the decrease, parameter effects of the reform remain positive. The inclusion of additional controls (time-varying marital status of mother, male partner’s educational attainment, and macroeconomic characteristics) in models M3 and M4 does not bring about any substantial change in the results.

**Table 2:** Exponentiated coefficients of the policy variable for progression to second and third birth (mixture cure model), women born 1960–1999, Estonia

	Policy variable			
	Model M1	Model M2	Model M3	Model M4
*Parity progression 1 → 2*				
Incidence submodel	1.96***	1.28***	1.26***	1.25***
Duration submodel	0.91***	0.87***	0.87***	0.88***
*Parity progression 2 → 3*				
Incidence submodel	1.74***	1.33***	1.31***	1.34***
Duration submodel	1.19***	0.84***	0.84***	0.85***

Model M1 includes the process time and the policy variable

Model M2 includes, in addition to variables in Model M1, controls for women’s age at previous birth, women’s partnership status at previous birth, sex of previous child(ren), women’s ethnicity, women’s place of residence at previous birth, women’s educational attainment at previous birth, women’s occupational status at previous birth, and women’s birth cohort

Model M3 includes, in addition to variables in Model M2, controls for educational attainment of the father of previous child, and mother’s time-varying marital status

Model M4 includes, in addition to variables in Model M3, GDP growth rate, unemployment rate, and consumer confidence index

*Source:* Statistics Estonia, authors’ calculations

^†^p < .1, *p < .05, **p < .01; ***p < .001

To allow for an interpretation of the estimates in terms of parity progression, we calculated difference in predicted probabilities of the second and third births associated with the policy variable. The results show that in the non-adjusted incidence model (M1), the policy reform is associated with a 11.0 percentage point increase in the probability of second birth, relative to the pre-reform period. In the final model (M4), the difference in the probability of second birth is 5.5 percentage points. For third birth, differences in the predicted probability are somewhat larger (13.8 percentage points) in the non-adjusted incidence model, but after adjusting for the effects of control variables, the parental leave reform makes a smaller difference for the probability of third births. In the final model, it is reduced to 3.7 percentage points.

It is important to note that odds ratios and predicted probabilities create a different impression of parental leave reform on the quantum of second and third births. The change in odds ratios, associated with the reform, is larger for third births, while the increase in predicted probability appears larger for second births. The difference stems from the fact that the odds ratios do not take into account an approximate twofold difference in the proportion of one- and two-child mothers who have another child. Therefore, probabilities must be preferred to odds ratios for obtaining an account of changes in fertility quantum, comparable across parities.

Results obtained from the duration submodel show differences in the tempo of childbearing. More specifically, the exponentiated parameter estimates indicate multiplicative effects of shortening (exponentiated coefficient under 1) or lengthening (over 1) of the time from previous birth to conception of another child. With the exception of non-adjusted model for third births, all duration submodels in Table 2 indicate that the parental leave reform is associated with a sooner conception of the next child. The estimates based on the final model (M4) show that in the period after the parental leave reform, the time to the conception of the second child is reduced by a factor of 0.88, or by 12% compared to the pre-reform period. For third births, the shortening effect is even more pronounced (15%).

#### Estimates for Control Variables

The estimates for control variables are shown in Table 3. Age of women at previous birth is associated with a lower propensity of having another child, as well as shorter birth intervals. The latter finding reflects the simple fact that giving birth at a later age leaves less time for having another child.

**Table 3:** Exponentiated coefficients of control variables for progression to second and third birth (mixture cure model), women born 1960–1999, Estonia

Control variable	Incidence submodel		Duration submodel	
	Parity progression			
	1 → 2	2 → 3	1 → 2	2 → 3
*Woman’s age at previous birth*				
Under 20	9.92***	8.62***	1.52***	0.92
20–24	5.03***	5.06***	1.45***	1.21***
24–29	2.20***	2.39***	1.20***	1.32***
30–34	1	1	1	1
35–39	0.41***	0.45***	0.87***	0.66***
40–44	0.15***	…	0.86	…
*Woman’s partnership status at previous birth*				
Married	1	1	1	1
Cohabiting	1.36***	1.29***	0.84***	0.90***
Not partnered	0.82**	1.18†	0.92***	0.81***
*Woman’s marital status (time-varying)*				
Married	1	1	1	1
Never married	0.41***	0.98	1.51***	1.23***
Formerly married	0.34***	0.95	1.50***	1.03
Re-married	4.22***	3.55***	0.58***	0.59***
*Sex of previous child(ren)*				
Boy	1	–	1	–
Girl	0.96†	–	1.02**	–
Boy and girl	–	1	–	1
Both girls	–	1.20***	–	0.89***
Both boys	–	1.22***	–	0.89***
*Woman’s education at previous birth*				
Low (ISCED0–2)	0.89*	1.15**	0.84***	0.75***
Medium (ISCED3–4)	1	1	1	1
High (ISCED5–8)	1.44***	1.35***	0.94***	1.06*
*Male partner’s education at previous birth*				
Low (ISCED0–2)	0.92†	1.25***	0.94***	0.86***
Medium (ISCED3–4)	1	1	1	1
High (ISCED5–8)	1.37***	1.32***	0.87***	0.95*
*Woman’s occupation/activity status at previous birth*				
Upper white collar	1	1	1	1
Lower white collar	0.91**	0.92†	1.08***	1.09**
Manual occupation	0.83***	0.99	0.99	1.03
Unemployed	0.84**	1.06	0.97	0.92*
Homemaker	0.78***	1.13*	0.98	0.92**
Student	1.42***	1.44**	1.17***	0.92†
Other	0.42***	1.58*	0.79**	0.97
*Woman’s ethnicity*				
Estonian	1	1	1	1
Other ethnic groups	0.50***	0.54***	1.45***	1.12***
*Woman’s area of residence at previous birth*				
Capital	1	1	1	1
Areas surrounding the capital	1.21***	0.99	0.91***	1.01
Big towns	1.17***	1.02	0.95***	0.99
Smaller towns and rural areas	1.19***	1.04	0.85***	0.89***
*Macroeconomic controls (time-varying)*				
GDP growth rate	0.89***	0.86***	0.98***	0.97**
Unemployment rate	1.16***	1.17***	1.05***	1.04***
Consumer confidence index	1.35***	1.40***	1.12***	1.14***
Number of women	125,284	89,936	125,284	89,936

The estimates for woman’s birth cohort and for women with missing values for each covariate are not shown. The estimates for the main explanatory variable are presented in Table 2

*Source:* Statistics Estonia, authors’ calculations

^†^p < .1, *p < .05, **p < .01; ***p < .001

Relative to the reference group (married women), women who had been cohabiting at previous birth show a higher odds ratio of having another child, as well as shorter birth intervals. This reflects a widespread acceptance of unmarried cohabitation as a context for having children in the study population (Puur et al., 2012; Sakkeus et al., 2019). Somewhat surprisingly, women who were not partnered at previous birth exhibit higher odds of third births, and experience subsequent birth sooner than their married counterparts. This may be due to the fact that our partnership variable reflects the situation at previous birth; it does not consider (higher-order) partnerships that have started after that time.

Never- and formerly married women show lower propensity of second birth and longer time to conception compared to the reference group (first-time mothers in first marriage). By contrast, the odds of third birth are not significantly lower for never- and formerly married women. Differences in the length of third-birth intervals are also smaller than those for second births, with only never married exhibiting slower progression to the next parity than the reference group. Smaller differences between marital status groups in the transition to third birth can be explained by the higher proportion of third children who are born in second or higher-order unions. Research into partnership dynamics in the study population has shown that higher-order unions are less frequently converted into marriage (Rahnu et al., 2015). Women in these unions appear as never- or formerly married in our data. Finally, the odds of second or third birth are very high among women in their second or higher-order marriage; the transition to the next child also is much faster in this group. This evidently reflects women’s wish to consolidate their new marriage by having a common child.

The effect of sex preference is evident in the transitions to both second and third parity. For second births, having a girl as the first child is associated with slightly lower odds of second birth and longer birth intervals. For third births, both submodels reveal a preference for the mixed subset.

Educational attainment also exhibits different patterns for second and third births. For second births, the incidence submodel shows a positive gradient, which corroborates previous studies from the same setting (Klesment & Puur, 2010). For third births, the relationship follows a U-shaped pattern, with the lowest odds ratio for women with medium education. Unlike most control variables, education does not show similarity of the patterns revealed by the incidence and duration submodels. For instance, women with high education exhibit the highest odds ratio of third birth, but have longer birth intervals than their peers.

Lower occupational status, unemployment or being homemakers, or belonging to a small residual category are all associated with lower odds of second birth, compared to the reference group (women in upper white-collar occupations). For third births, the pattern is reversed for homemakers and the residual category, and no significant difference from the reference group in the odds of having another child can be observed for women in manual occupations or who are unemployed. Somewhat unexpectedly, elevated odds of having another child are characteristic of women who had been in education at the time of their previous birth. This can be explained by the fact that most of these women will attain high education, which is associated with higher odds of second and third births in the study population. Similar to education, the patterns for occupational/activity status do not fully match in incidence and duration submodels.

People of other ethnic backgrounds than Estonians (mostly Russians and Russian-speakers) exhibit a lower propensity of having a second and third child, as well as longer birth intervals. This is in accord with previous research, which has shown that fertility behaviour of migrants from Russia to Estonia and their descendants exhibits similarity to that of the sending population (Puur et al., 2017, 2019). Living in the capital is associated with both a lower propensity to have a second birth and longer birth intervals, while differences between other areas are statistically insignificant. In the progression to the third birth, regional differences are insignificant, with the exception of shorter birth intervals in small towns and rural areas.

The relationship between our three macroeconomic indicators and the odds of second and third births is not uniform. The consumer confidence index shows a pro-cyclical pattern that is usually found for macroeconomic variables (Sobotka et al., 2011). We think that the absence of a pro-cyclical association for the GDP growth and unemployment rate can be explained by the use of mixture cure models, which separate the effect of the covariates on the quantum and tempo of childbearing. Although the pro-cyclical relationship is not found for the GDP growth and unemployment rate in the incidence submodel, it is found for these covariates in the duration submodel. Higher GDP growth is associated with shorter birth intervals, while higher unemployment is associated with longer time to the event. In other words, our findings suggest that fertility declines during recession may be to a significant extent due to postponement of childbearing. This in line with the notion that macroeconomic conditions influence especially the timing of childbearing, and in most cases do not leave an imprint on cohort fertility levels (Sobotka et al., 2011).

## Summary and Discussion of the Findings

This study has investigated the relationship between generous earnings-related parental leave and fertility behaviour.

The findings support our first hypothesis (H1) that the speed premium feature of the Estonian parental leave is associated with accelerated transition to the next birth. Interestingly, the shift towards a closer spacing of births did not fully manifest itself immediately after the reform, but took five years to build up, presumably due to adjustments in women’s labour market behaviour, and a stepwise extension of the duration of the leave. Although closer spacing of births in response to speed premium has been found in previous studies (Andersson, 1999; Hoem, 1990, 1993; Matysiak & Szalma, 2014; Št’astná & Sobotka, 2009), the gradual nature of the change has not received much attention. However, recognising this possibility is important because the effects that build up over a longer time may go understated or overlooked in models based on, for example, the regression discontinuity design.

Our results also suggest that the change in child spacing generated by the policy reform is not a short-lived phenomenon. After a temporary decrease caused by the Great Recession, fertility rates at short durations returned to the high levels characteristic of pre-recession parity cohorts. This is at odds with recent findings from Sweden, where the previously documented short birth intervals have become increasingly less common since the mid-1990s (Miranda, 2020). Conflicting results warrant further research in other settings where parental leave schemes have provided speed premium for a longer time.

Following our second hypothesis (H2), we expected that the introduction of generous parental leave would increase fertility quantum. The results seem to support the hypothesis. The Kaplan–Meier estimates show that the exposure to policy reform was associated with elevated parity progression ratios 10 or more years after the previous birth. The mixture cure models show increased propensity in having an additional child, associated with our policy variable. Claims that a policy reform has a positive impact on fertility quantum are sometimes questioned on the grounds that the reform is not accompanied with increase in cohort fertility (Sobotka et al., 2020). In Estonia, cohort fertility has recovered among women exposed to the new parental leave system in their prime childbearing years. After a decrease in cohorts born in the 1960s, cohort fertility increased among women born in the 1970s. The number of children ever born increased from 1.83 in the 1970 cohort to 1.91 among women born in 1977–1978. The increase was caused by a higher progression to second and third births, which more than compensated an increase in childlessness and a decrease in higher-order births (Statistics Estonia, 2022).

While the current research design cannot give an absolute guarantee that the policy reform was the cause of the observed increase in fertility quantum, several arguments support the causal interpretation. First, the universality of parental leave in our study population implies that the results are not biased by selective policy uptake (Neyer & Andersson, 2008). Second, the reform investigated in this study constituted a clear one-off break with the previous system. It was not intertwined with other major changes in family policies (Puur & Vseviov, 2019). Third, there is coherence between the results from the descriptive analysis and mixture cure models and the patterns revealed during the analysis. For instance, the progression to next birth in the pre-reform parity cohorts (a close similarity at the time when cohorts were not exposed to policy change, followed by divergence exactly at time when the cohorts became exposed) is well in line with assumption of parallel trends, the main assumption in the difference-in-differences approach (Angrist & Pischke, 2009).

Our third hypothesis (H3) posited that changes in both the quantum and tempo of childbearing associated with the parental leave reform are more pronounced in the transition to second birth. The findings on fertility quantum are in line with this assertion. However, at odds with the expectation, the shift towards shorter birth intervals is more extensive for third births. This finding can be explained by the fact that women at risk of a third birth are older on average and have less time to realise their childbearing plans than their counterparts at risk of a second birth, which may have led to a more extensive compression of birth intervals among them. This explanation is indirectly supported by the results obtained from the duration submodel for control variables, as a woman’s higher age at the beginning of the birth interval exhibits a stronger effect (in older age groups) for third births.

This study is not without limitations. Although we used a large number of control variables, the individual-level controls were mostly time-fixed, measured at the beginning of birth intervals. Some of these characteristics, such as occupation/activity status and area of residence, may be quite variable over an individual’s life course. That said, it seems unlikely that the effects of the parental leave reform on the tempo and quantum would be markedly altered if we had been able to account for changes in all individual characteristics. The inclusion of time-varying marital status in the model, in addition to partnership status at the beginning of birth interval, resulted only in a marginal change in the effects of policy variable. Another limitation is that this study did not investigate variation in the effects of parental leave reform between subgroups of women, documented in previous research (Andersson et al., 2006; Cannonier, 2014; Cygan-Rehm, 2016; Võrk et al., 2009). Nor did our study address the effect of leave taking on parents’ subsequent labour market position and earnings (Budig et al., 2012; Evertsson & Duvander, 2011; Lalive & Zweimüller, 2009; Pettit & Hook, 2005). We expect that some of these issues can be addressed in our future research.

Notwithstanding these limitations, we can draw some important conclusions from this study. First, our findings suggest that the effects of well-paid parental leave are not limited to birth spacing, but can also affect fertility quantum. Our study thus supports a recent reappraisal of the impact of parental leave reforms by Bergsvik et al. (2021), who concluded that generous job-protected parental leave has larger effects on fertility than previously thought, particularly when it is combined with improvements in the availability of public childcare. Second, our results draw attention to several key elements that seem to be important for parental leave to have a positive impact on fertility levels. In addition to the number of benefits, the possibility to retain the level of benefits (if births are spaced closely) and sufficient duration of leave that allows parents to take advantage of the speed premium, all seem to matter. An improved understanding of the role of each of these elements calls for more research on countries that have earnings-related parental leave schemes, ideally in the framework of comparative studies using approaches that allow causal inference. It is also important to expand the analysis to the effects of earnings-related parental leave on first births, which is a topic rarely discussed in the literature.

## Data Availability

The data that support the findings of this study are available from Statistics Estonia but restrictions apply to the availability of these data, which were used under licence for the current study, and so are not publicly available.

## Footnotes

1 Parental leave schemes may also influence fertility by redistributing the time cost of childrearing between parents. In the gender revolution framework (Goldscheider et al., 2015), increasing father involvement in childrearing is expected to reduce the mother’s opportunity costs, potentially increasing (her) fertility. However, research into the effects of leave sharing is beyond the scope of our study.

2 The Generations and Gender Survey reveals that among women aged 20–39 in Estonia, the intended parity progression ratio from first to second child was 78% in 2004. The cohort parity progression ratio among women born in the 1960s was 70%. For the transition to third birth, the gap between intended and actual parity progression ratios was much smaller.

3 In the models for third births, we have censored observations on 1 July 2017, in order to avoid confounding influence of the reform of child benefits in 2017, during which benefits for families with three or more children were increased particularly strongly (Puur & Vseviov, 2019, p. 410; Sinisaar 2021).

4 The increase of third-birth rate in the 2015 cohort, 31–36 months since the previous birth, is caused by a marked increase in child benefits for families with three or more children, which took place in 2017.

5 For readability, the curves are presented for a selection of parity cohorts from 1996 to 2014. In most cases, the curves for the omitted cohorts lie between those of the neighbouring cohorts shown in the figure.

6 For simplicity, we prefer to talk about second and third births when discussing results.
